# Supplementary material for: Complexome profiling of the Chlamydomonas psb28 mutant reveals TEF5 as an early PSII assembly factor
Source: Plant Cell. 2025 Mar 17;37(6):koaf055. doi: 10.1093/plcell/koaf055 (PMC12164590; doi:10.1093/plcell/koaf055)
Supplement: koaf055_Supplementary_Data [file koaf055_supplementary_data.zip › psb28_interactive.zip!/plots/intensity/qualityPlots/peptides/SULP1_Cre07.g348600.t1.1.html]

### Cre07.g348600.t1.1

Trivial name: SULP1  
SeqCoverage [median]: 5.60  
Mass: 43.71 kDa  
  
 

| Sequence | MissedCleavage | Mass [Da] | wtIntensity | muIntensity | Score |
| --- | --- | --- | --- | --- | --- |
| ASLVPLNVFIAR | 0 | 1298.77 | 2066.59 | 0.00 | 76.68 |
| TMQPVMQEIQK | 0 | 1331.66 | 0.00 | 473.91 | 64.30 |
|  |  |  | 2066.6 | 473.9 |  |
